# Stimulation and inhibition of enzymatic hydrolysis by organosolv lignins as determined by zeta potential and hydrophobicity

**DOI:** 10.1186/s13068-017-0853-6

**Published:** 2017-06-24

**Authors:** Yang Huang, Shaolong Sun, Chen Huang, Qiang Yong, Thomas Elder, Maobing Tu

**Affiliations:** 1 College of Chemical Engineering, Nanjing Forestry University, Nanjing, 210037 China; 2 Department of Biomedical, Chemical and Environmental Engineering, University of Cincinnati, 2901 Woodside Drive, Cincinnati, OH 45221 USA; 3 USDA-Forest Service, Southern Research Station, 521 Devall Drive, Auburn, AL 36849 USA

**Keywords:** Ethanol organosolv lignin, Enzymatic hydrolysis, Hydrophobicity, Zeta potential, NMR

## Abstract

**Background:**

Lignin typically inhibits enzymatic hydrolysis of cellulosic biomass, but certain organosolv lignins or lignosulfonates enhance enzymatic hydrolysis. The hydrophobic and electrostatic interactions between lignin and cellulases play critical roles in the enzymatic hydrolysis process. However, how to incorporate these two interactions into the consideration of lignin effects has not been investigated.

**Results:**

We examined the physicochemical properties and the structures of ethanol organosolv lignins (EOL) from hardwood and softwood and ascertained the association between lignin properties and their inhibitory and stimulatory effects on enzymatic hydrolysis. The zeta potential and hydrophobicity of EOL lignin samples, isolated from organosolv pretreatment of cottonwood (CW), black willow (BW), aspen (AS), eucalyptus (EH), and loblolly pine (LP), were determined and correlated with their effects on enzymatic hydrolysis of Avicel. EOLs from CW, BW, and AS improved the 72 h hydrolysis yield by 8–12%, while EOLs from EH and LP decreased the 72 h hydrolysis yield by 6 and 16%, respectively. The results showed a strong correlation between the 72 h hydrolysis yield with hydrophobicity and zeta potential. The correlation indicated that the hydrophobicity of EOL had a negative effect and the negative zeta potential of EOL had a positive effect. HSQC NMR spectra showed that β-*O*-4 linkages in lignin react with ethanol to form an *α*-ethoxylated β-*O*-4ʹ substructure (Aʹ) during organosolv pretreatment. Considerable amounts of C_2,6_–H_2,6_ correlation in *p*-hydroxybenzoate (PB) units were observed for EOL–CW, EOL–BW, and EOL–AS, but not for EOL–EH and EOL–LP.

**Conclusions:**

This study revealed that the effect of lignin on enzymatic hydrolysis is a function of both hydrophobic interactions and electrostatic repulsions. The lignin inhibition is controlled by lignin hydrophobicity and the lignin stimulation is governed by the negative zeta potential. The net effect of lignin depends on the combined influence of hydrophobicity and zeta potential. This study has potential implications in biomass pretreatment for the reduction of lignin inhibition by increasing lignin negative zeta potential and decreasing hydrophobicity.

**Electronic supplementary material:**

The online version of this article (doi:10.1186/s13068-017-0853-6) contains supplementary material, which is available to authorized users.

## Background

Lignin from different biomass sources and pretreatment methods have shown distinct effects on enzymatic hydrolysis of lignocellulosic substrates. Lignin content has been often negatively correlated with the hydrolysis yield of pretreated biomass [1, 2]. Good association was observed between the digestibility of ionic liquid-pretreated maple wood and its lignin content [3]. However, neutral and positive effects of lignin on enzymatic hydrolysis of lignocellulose have also been reported recently [4, 5]. Lignin-rich residue from dilute acid-pretreated switchgrass showed no inhibition on cellulose saccharification [6]. Extractable lignin from organosolv-pretreated sweetgum-enhanced enzymatic hydrolysis [7]. Ethanol organosolv lignin (EOL) from hardwood has been observed to improve the 72 h hydrolysis yield of Avicel. Similar effects have been reported for modified lignins, such as lignosulfonates, ɤ-carboxylated, and hydroxypropylated lignin [5, 8, 9].

Non-productive binding has been suggested as one of the main reasons for lignin inhibition [10], which reduces the availability and activity of the cellulase enzymes. Hydrophobic interactions were reported to be the leading attractive force between cellulase and lignin [11, 12], which was revealed by atomic force microscopy between specialized hydrophobic tips and immobilized enzymes and comparing tips with OH and COOH groups [11]. The hydrophobicity of the cellulase enzyme surface has also been calculated using an estimation of the clustering of non-polar atoms and it was suggested that hydrophobic interactions drive enzyme adsorption onto lignin [13]. Phenolic OH groups have been suggested to mediate the lignin inhibition, which was revealed by lignin hydroxypropylation [9]. Recently, condensed syringyl and guaiacyl phenolic units have been proposed to be responsible for lignin inhibition, in which the condensed aromatic rings enhance the hydrophobic interactions and the phenolic OH groups boost the hydrogen bonding between enzymes and lignin [14]. However, electrostatic interactions also played an important role in between cellulase and lignin [15–17]. Carboxylic acid group in lignin has been reported to contribute to the increase of hydrophilicity and negative charge of lignin, which may decrease the non-productive binding and enhance the enzymatic hydrolysis [8]. Lignosulfonate was found to enhance cellulose conversion by enlarging electrostatic repulsion and weakening the non-productive binding of cellulase to lignin [5]. The electrostatic interactions between cellulase and lignin were also examined by changing the pH of the hydrolysis buffer and the observation of a significant increase of unabsorbed enzyme in the supernatant when the pH increased [17]. However, the hydrophobic interactions could interfere with electrostatic interactions between cellulase and lignin. The incorporation of two interactions at the same time into the consideration of lignin effects on enzymatic hydrolysis has not been investigated, but is critically needed to better understand the mechanism of lignin inhibition and stimulation.

Lignin hydrophobicity and zeta potential essentially are governed by lignin structures and functional groups. The lignin polymer is composed of three primary units: *p*-hydroxyphenyl (H), guaiacyl (G), and syringyl (S) [18]. Li et al. found that with hot-water pretreatment an *Arabidopsis* mutant containing mainly S-rich lignin showed higher yield of released sugars than wild-type and G-rich plant [19]. The lignin units are linked together by C–C and aryl-ether bonds with a few functional groups including—OCH_3_ groups, phenolic OH groups, and carbonyl (C=O) groups. Most of these lignin linkages and functional groups can be identified by HSQC NMR or ^31^P NMR [14, 20].

The objective of this study is to elucidate the relationship between physicochemical properties of EOL lignins and their inhibition and/or stimulation on enzymatic hydrolysis. Previous research has suggested that certain EOL lignins should be preserved in pretreated biomass and solvent washing after organosolv pretreatment could be eliminated [21]. Therefore, it is important to understand why certain EOL lignins can stimulate enzymatic hydrolysis. EOL lignins were isolated by organosolv pretreatments of hardwoods and softwoods. Their inhibitory and stimulatory effects on enzymatic hydrolysis of pure cellulose (Avicel) were correlated with the hydrophobicity and zeta potential of lignins. It is hypothesized that the effect of EOL lignins on enzymatic hydrolysis is a function of two factors. Inhibition by EOL lignins is controlled by lignin hydrophobicity and the stimulation is governed by the negative zeta potential. Cellulase distribution during enzymatic hydrolysis was determined to examine whether the addition of lignins can increase or decrease the free cellulase in solution. Langmuir adsorption isotherms were used to measure the adsorption affinity of enzymes to isolated lignins. Scanning electron microscopy (SEM) and HSQC NMR were applied to show the micromorphology and structural features of the isolated lignins.

## Results and discussion

### Inhibitory and stimulatory effects of organosolv lignins on enzymatic hydrolysis of Avicel and substrates

**Fig. 1:**
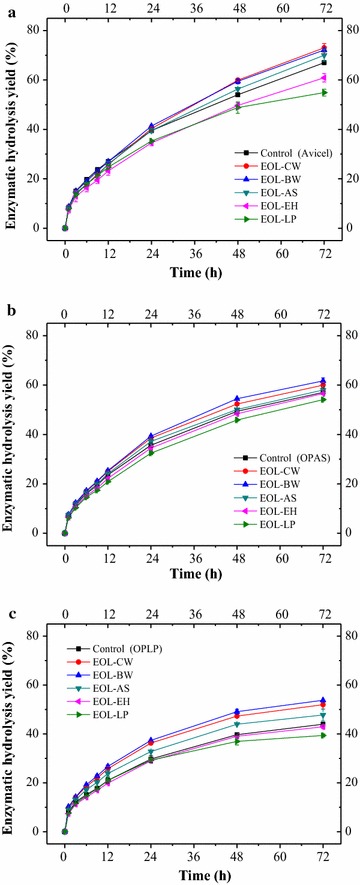
Effect of the addition of EOL lignins on enzymatic hydrolysis of Avicel and pretreated substrates. **a** Avicle; **b** OPAS and **c** OPLP

To investigate the distinctive effect of hardwood organosolv lignins on enzymatic hydrolysis, five organosolv lignins (EOL–CW, EOL–BW, EOL–AS, EOL–EH, and EOL–LP) were added to the enzymatic hydrolysis of Avicel and their individual effects on the 72 h hydrolysis yield were compared (Fig. 1a). It was observed that three hardwood organosolv lignins (EOL–CW, EOL–BW, and EOL–AS) increased the 72 h hydrolysis yield of Avicel, and two lignins (EOL–EH and EOL–LP) decreased the 72 h hydrolysis yield. The negative effect of EOL–EH (eucalyptus, hardwood) was unexpected. Specifically, the addition of EOL–CW, EOL–BW, and EOL–AS enhanced the 72 h hydrolysis yield from 65.00 to 73.02, 72.15, and 70.04%, respectively. In contrast, EOL–EH and EOL–LP decreased the 72 h hydrolysis yield from 65.0 to 60.90 and 54.92%, respectively. The initial hydrolysis rate (1.11 g/L/h) was least affected by the addition of organosolv lignins. In addition, enzymatic hydrolysis of organosolv-pretreated aspen (OPAS) and loblolly pine (OPLP) with the addition of lignins were also evaluated (Fig. 1b, c). For the hydrolysis of OPAS, the addition of EOL–CW, EOL–BW, and EOL–AS increased the 72 h hydrolysis yield from 57.0 to 60.0, 61.7, and 58.0%, respectively (Fig. 1b). Conversely, the addition of EOL–EH and EOL–LP decreased the 72 h hydrolysis yield to 56.6 and 54.1%, respectively. For the hydrolysis of OPLP, the addition of EOL–CW, EOL–BW, and EOL–AS increased the 72 h hydrolysis yield from 44.0 to 52.0, 53.8, and 47.8%, respectively (Fig. 1c), while, the addition of EOL–EH and EOL–LP decreased the 72 h hydrolysis yield to 42.9 and 39.4%, respectively. The results agreed well with previous findings in which EOL–SG from sweetgum (4 g/L) increased the 72 hydrolysis yield of Avicel by 7% and EOL–LP decreased the hydrolysis yield by 9% under 5 FPU of Novozym 22C [4]. In addition, different concentrations of EOL–AS and EOL–LP (2, 4, and 8 g/L) have been added into the enzymatic hydrolysis of Avicel (Additional file 1: Figure S1). Higher concentration of EOL–AS resulted in higher 72 h hydrolysis yield, but the difference was only 3%. On the contrary, the higher concentration of EOL–LP decreased the 72 h hydrolysis yield significantly from 62.0% (with 2 g/L EOL–LP) to 39.6% (with 8 g/L EOL–LP).

Similar results have been reported on the effects of lignosulfonate on enzymatic hydrolysis of sulfite-pretreated biomass [5, 22], in which it was found that lignosulfonate increased the 72 h hydrolysis yield of sulfite-pretreated poplar from 41.6 to 65.7%. Lignosulfonate has been suggested to reduce non-productive binding between enzymes and residual lignin due to the electrostatic repulsion of negative-charged lignosulfonate groups [5]. In this study, pure cellulose substrate Avicel was used, such that the enhancement most likely is coming from the decrease of non-productive binding between enzymes and cellulose. Previous research suggested that cellobiohydrolases (Cel7A) would bind on cellulose with two binding modes (tight binding and weaker binding) [23]. They hypothesized that the cellulose binding module (CBM) binding to the hydrophobic part of cellulose would produce non-productive complexes. We believe that EOL lignins in this study can reduce the formation of non-productive complexes through electrostatic repulsion. It should be noted that organosolv lignin (EOL–EH) from one of the hardwoods (eucalyptus) was unexpectedly observed to be negative on enzymatic hydrolysis. We believe this was related to the specific physicochemical properties (such as hydrophobicity, zeta potentials, and functional groups) of EOL–EH. Previously, lignin has been reported to boost the activity of cellulose oxidizing enzyme lytic polysaccharide monooxygenase (LPMO) in Cellic CTec2, which was used in this study [24, 25]. This might be used to explain the positive effects of EOL–AS, EOL–BW, and EOL–CW. However, the role of lignin in LPMO cannot explain the negative effects of EOL–EH and EOL–LP. Therefore, the positive and/or negative effects of EOL lignin are more likely resulting from the combinational influence of lignin hydrophobicity, zeta potential, and functional groups.

### Effects of organosolv lignins on cellulase distribution during enzymatic hydrolysis of Avicel

**Fig. 2:**
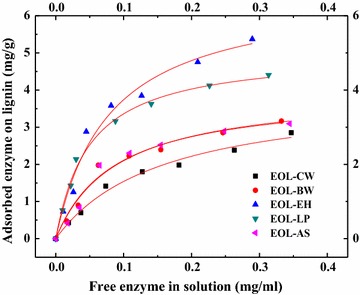
Effect of EOL lignins on cellulase distribution during enzymatic hydrolysis of Avicel

To examine whether the addition of organosolv lignins can reduce the non-productive binding between enzymes and cellulose, the free cellulase enzyme (protein) concentrations were determined during the 72 h hydrolysis of Avicel **(**Fig. 2). The results showed that the addition of EOL–CW, EOL–BW, and EOL–AS increased the free cellulase enzymes in solution, but EOL–EH and EOL–LP reduced the free cellulase enzymes significantly in solution. Specifically, for the enzymatic hydrolysis of Avicel, the free cellulase enzymes percentage decreased to 69.0% at 72 h. The addition of EOL–CW, EOL–BW, and EOL–AS increased the free enzyme percentage to 88.4, 82.4, and 80.0% at 72 h, respectively. The increase of free cellulase enzymes by 16–28% could be the main reason for the positive effects of these three organosolv lignins. On the contrary, the addition of EOL–EH and EOL–LP decreased the free enzymes percentage to 35.5 and 12.2% at 72 h, respectively. A strong correlation was observed between the 72 h hydrolysis yield and the free enzymes percentage (*y* = −0.2335*x* + 75.609, *r* ^2^ = 0.994). This indicated that the positive effects of EOL lignins were related to their influence on cellulase distribution in hydrolysis, which resulted in the decrease of non-productive binding between enzyme and cellulose. While the negative effects of EOL–EH and EOL–LP reduced the free cellulase enzymes due to the increase of the non-productive binding between enzyme and EOL lignins.

### Langmuir adsorption isotherms between organosolv lignins and cellulase enzymes

**Fig. 3:**
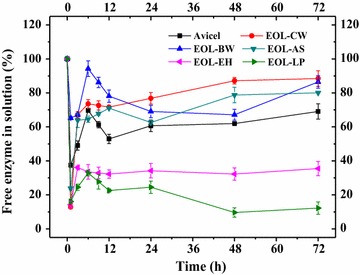
Cellulase enzyme adsorption on EOL lignins

To explore why EOL–EH decreased the 72 h hydrolysis yield and free cellulases during the hydrolysis of Avicel, Langmuir adsorption isotherms of cellulase enzymes onto EOL–CW, EOL–BW, EOL–AS, EOL–EH, and EOL–LP were determined and compared (Fig. 3). EOL–EH and EOL–LP showed two- to threefolds higher in Langmuir constants (*K*) and distribution coefficients (*R*) than those from EOL–CW, EOL–BW, and EOL–AS (Table 1). Specifically, EOL–EH (*K* = 12.753 mL/mg, *R* = 0.085 L/g) displayed similar high binding strength as EOL–LP (*K* = 20.157 mL/mg, *R* = 0.101). EOL–BW (*K* = 9.347 mL/mg, *R* = 0.046 L/g) and EOL–AS (*K* = 11.677 mL/mg, *R* = 0.046 L/g) presented comparable low binding strength. EOL–CW showed the lowest Langmuir constant and distribution coefficient (*K* = 6.367 mL/mg, *R* = 0.025 L/g). This corresponded well with the highest improvement of hydrolysis by the addition of EOL–CW (Fig. 1). Distribution coefficient (*R* = *K* × *Γ*_m_) has been often used to evaluate the binding strength of lignin with cellulase enzymes [4, 26]. A strong negative association was observed between the 72 h hydrolysis yield and the binding strength (*y* = −2.84*x* + 0.85, *r* ^2^ = 0.964).

**Table 1:** Langmuir adsorption isotherm parameters from enzyme adsorption on lignins

Cellulases	*Г*_max_ (mg/g)	*K* (mL/mg)	*R* (L/g)
Cellulases on EOL–EH	6.672	12.753	0.085
Cellulases on EOL–LP	5.026	20.157	0.101
Cellulases on EOL–BW	4.900	9.347	0.046
Cellulases on EOL–CW	3.960	6.367	0.025
Cellulases on EOL–AS	3.919	11.677	0.046

In this study, it appeared that the EOL–EH and EOL–LP lignins with higher binding strength (*R*) resulted in an inhibitory effect, while EOL–CW, EOL–BW, and EOL–AS with lower binding strength (*R*) resulted in stimulatory effect. Similar observations have been recently reported for the inhibitory effect of EOL lignins from lodgepole pine and hybrid polar on the enzymatic hydrolysis of Avicel [26], in which they found that EOL lignin (LPP1) with the highest binding strength (3.274 L/g) showed strongest inhibition and dropped the 48 h hydrolysis yield from 60 to 12%. The negative effect of EOL–EH and EOL–LP was probably due to a considerable amount of enzymes that were bound non-productively with lignin. The tightly bound enzymes were most likely denatured and reduced the available enzymes for hydrolysis. It was unexpected that the EOL lignins with lower binding strength (*R*) resulted in an increase of free enzymes and 72 h hydrolysis yield. This indicated that the weakly bound enzymes were not denatured and can be desorbed from lignin. However, the increase in free enzymes in the presence of lignins with low *R* value is not fully understood. We believe that another parameter (zeta potential) is involved in the increase of free enzymes in these lignins with low *R* values.

### Zeta potential and hydrophobicity analysis of organosolv lignins

To examine which driving force is responsible for increasing free enzymes by EOL–CW, EOL–BW, and EOL–AS, zeta potential and hydrophobicity of five lignins were measured and compared (Table 2). The results showed that the zeta potential of all five lignins was negative, and the EOL lignins that increased the free enzymes displayed twofold higher zeta potential than those lignins that decreased free enzymes. Specifically, zeta potentials of EOL–CW, EOL–BW, and EOL–AS were −15.30, −15.77, and −13.27 mV, respectively, and zeta potentials of EOL–EH and EOL–LP were −8.37 and −6.42 mV, respectively.

**Table 2:** Hydrophobicity, zeta potential, and particle size of the isolated lignins

Lignin sample	Particle size (μm)	Hydrophobicity (L/g)	Zeta potential (mV)	72 h hydrolysis yield (%)
EOL–AS	0.75	0.43	−13.27	70.04
EOL–CW	0.62	0.51	−15.30	73.02
EOL–BW	0.59	0.80	−15.77	72.15
EOL–EH	0.28	0.80	−8.37	60.90
EOL–LP	0.23	1.11	−6.42	54.92

Cellobiohydrolase I (CBH I or Cel7A) is one of the major components (60%) in *Trichoderma reesei* enzyme cocktail [27]. The pKa of cellobiohydrolase I has been reported as 4.3 [13], such that cellobiohydrolase I is negatively charged under pH 4.8. So, the higher negative zeta potential of EOL–CW, EOL–BW, and EOL–AS yielded stronger electrostatic repulsion between lignin and enzymes and reduced the non-productive binding between enzymes and Avicel. A similar observation was reported for the lignosulfonate and its mitigation of non-productive binding [5]. Similar effects have been observed on the isolated lignin from steam and organosolv-pretreated biomass [8], in which they found that carboxylic acid group in lignin might reduce the negative effects of lignin on enzymatic hydrolysis. It should be noted that zeta potential of Avicel was determined to be −0.20 mV. Thus, no repulsion existed between cellulase enzymes and Avicel. In this study, EOL–EH and EOL–LP showed lower zeta potential (<−8.4 mV). However, they could not yield adequate electrostatic repulsion to increase free enzymes during the hydrolysis. We hypothesize that there is a balance between electrostatic repulsion and hydrophobic interaction, which probably is caused by zeta potential and the hydrophobicity of lignins, respectively. EOL–EH (0.80 L/g) and EOL–LP (1.11 L/g) showed higher hydrophobicity than EOL–CW (0.51 L/g) and EOL–AS (0.43 L/g) (Table 2), although EOL–EH and EOL–BW (0.80 L/g) had the same hydrophobicity. Hydrophobicity has been often characterized as a critical contributory factor to lignin inhibition of enzymatic hydrolysis [26]. Cellulase adsorption onto lignin by hydrophobic interactions has been suggested in steam-pretreated substrates [12]. In this study, the EOL–LP showed the highest hydrophobicity and resulted in the highest inhibition on enzymatic hydrolysis of Avicel. However, the EOL–BW with relatively high hydrophobicity showed a positive effect. This most likely was related to the higher zeta potential in EOL–BW. Therefore, we combined these two parameters into the regression analysis. The results showed strong correlation (*y* = 51.97 − 6.148 × hydrophobicity − 1.583 × zeta potential, adjust *r* ^2^ = 0.995) between the 72 h hydrolysis yield with hydrophobicity and zeta potential. The correlation indicated that the hydrophobicity was a negative effect and zeta potential was a positive effect. As a result, the effect of EOL lignins on enzymatic hydrolysis is a function of two factors. The stimulation or inhibition of EOL lignins will be dependent on lignin hydrophobicity and zeta potential. If hydrophobicity is the dominant factor, it will overcome the electrostatic repulsion and result in inhibition, such as EOL–EH and EOL–LP. If zeta potential is the major factor, it will overcome the hydrophobic interaction and result in stimulation, such as EOL–BW, EOL–CW, and EOL–AS. It should be noted that 72 h hydrolysis yield was also correlated well with the particle size (*r* ^2^ = 0.820). Table 2 shows that lignin hydrophobicity is negatively correlated lignin particle size. This correlation is understandable since surface area of lignin per unit mass is proportional to the radius of the particle. As a result, particle size of lignin could also play an important role in lignin effect.

### SEM analysis of organosolv lignins

To visualize surface morphology of lignins, SEM was used to characterize the five EOL lignins in this study (Additional file 1: Figure S2). EOL–BW, EOL–CW, and EOL–AS showed smooth and spherical shape of lignin particles. The average size of each particle was 0.59, 0.62, and 0.72 µm, respectively (Table 2, Additional file 1: Figure S3). The shape of EOL–EH was less spherical, and its size was much smaller (0.28 µm). EOL–LP showed irregular and flat shape of lignin with smallest size (0.23 µm). EOL–LP lignin particles appeared to aggregate more with each other. This indicated that EOL–LP was more hydrophobic and more inhibitory in enzymatic hydrolysis. It agreed well with our hydrophobicity results (Table 2).

## 2D HSQC NMR analysis of organosolv lignins

**Fig. 4:**
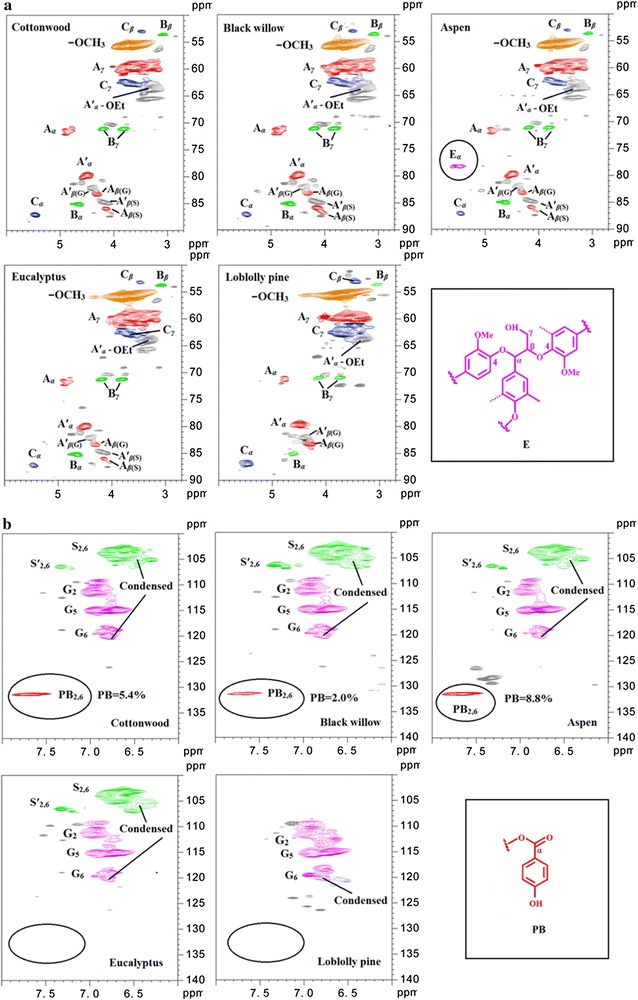
2D HSQC NMR spectra of the organosolv lignins. **a** Side-chain region and **b** aromatic region

To examine potential of lignin functional groups as factors in stimulatory or inhibitory effects, 2D HSQC NMR was used to characterize the five lignin samples (Fig. 4; Table 3).

**Table 3:** Quantitative analysis of the lignin fractions by integration of 2D HSQC NMR spectra (results expressed per 100 Ar)

Samples	S/G	β-*O*-4 (A)	β-*O*-4 (A′)	β–β	β-5	α-*O*-4/β-*O*-4	PB (%)
Cottonwood	1.56	1.0	14.0	6.9	3.3	ND	6.3
Black willow	2.69	3.0	10.3	7.8	1.8	ND	2.0
Aspen	2.35	0.8	9.5	4.9	1.4	3.6	8.8
Eucalyptus	4.91	0.8	12.2	10.1	1.5	ND	ND
Loblolly pine	ND	2.8	9.2	2.9	8.5	ND	ND

*ND* not detectable, *PB p*-hydroxybenzoate

HSQC NMR spectra showed similar cross peaks in both aromatic and side-chain regions for EOL–BW, EOL–CW, and EOL–AS, but were significantly different from EOL–EH and EOL–LP. This was consistent with their distinct effects on enzymatic hydrolysis. Specifically, the considerable amount of *p*-hydroxybenzoate lignin subunit (PB) at *δ*_C_/*δ*_H_ 131.3/7.62 ppm was found in EOL–CW, EOL–BW, and EOL–AS, but not observed in EOL–EH and EOL–LP [28, 29]. It is possible that the partial negative charge on the carbonyl oxygen (more accessible and no steric hindrance) in PB partially contributes to the negative potential of lignins. The 2D-HSQC spectra and other structures of the identified lignin sub-units are summarized in Fig. 4 [30, 31]. The interunit linkages of β-aryl-ether (β-*O*-4, *A*), resinol (β–β, *B*), phenylcoumaran (β-5, *C*), were identified by their cross peaks (Additional file 1: Figure S4, Table S1) at *δ*_C_/*δ*_H_ 71.9/4.88 (*A*_α_), 83.3/4.29 (*A*_β(G)_), 86.1/4.14 (*A*_β(s)_), 59.7/3.60 (*A*_γ_), 85.1/4.65 (*B*_α_), 53.5/3.04 (*B*_β_), 71.0/3.81 − 4.17 (*B*_γ_), 87.2/5.48 (*C*_α_), 53.0/3.45 (*C*_β_), and 62.7/3.71 (*C*_γ_), respectively. HSQC NMR spectra showed that β-*O*-4 linkages in lignin react with ethanol to form an *α*-ethoxylated β-*O*-4ʹ substructure (Aʹ). The methylene group of Aʹ *α*-OCH_2_CH_3_ was observed at δ_C_/δ_H_ 63.7/3.33 ppm. Ethyoxyl (–OCH_2_CH_3_) in Aʹ was also observed in EOL from *Buddleja davidii* [32]. The correlation of *C*_α_–*H*_α_ in Aʹ was observed at δ_C_/δ_H_ 79.9/4.50 ppm. The correlations of C_β_–H_β_ in Aʹ were observed at δ_C_/δ_H_ 82.2.9/4.39 ppm with G units and at δ_C_/δ_H_ 84.7/4.20 ppm with S units for EOL–CW, EOL–BW, EOL–AS, and EOL–EH. The relative abundances of the lignin interunit linkage were estimated from HSQC (Table 3). EOL–EH showed highest S/G ratio (4.9) among four hardwood EOL samples. β-*O*-4 substructure in A was low between 0.8 and 3.0% in all lignin samples, and ethoxylated β-*O*-4ʹ was considerably high in all lignins (9.2–14.0%). This indicated that significant amount of *β*-aryl-ether units (A) reacted at the *α* position with ethanol under acidic conditions.The ethoxyl group could potentially increase the lignin hydrophilicity and contribute to their stimulatory effect partially. EOL–EH showed the highest amount of β–β linkages (10.1%) and EOL–LP displayed the highest amount of β-5 linkages (8.5%), both of which could result from condensation reactions occurring in pretreatment process. This suggested that more condensation may have taken place in eucalyptus and loblolly pine pretreatment and resulted in more inhibition with of EOL–EH and EOL–LP.

The cross peaks in the aromatic region of HSQC showed well-separated signals of guaiacyl (G), syringyl (S), and *p*-hydroxybenzoate (PB) units of the lignins (Fig. 4). The correlations of C_2,6_–H_2,6_ in S units were observed at δ_C_/δ_H_ 103.7/6.68 ppm (S_2,6_) for EOL–CW, EOL–BW, EOL–AS, and EOL–EH. Small amounts of C_2,6_–H_2,6_ correlations in oxidized syringyl (Sʹ) were also observed at δ_C_/δ_H_ 106.5/7.35 ppm (Sʹ_2,6_) for all hardwood lignins. The G units showed 3 strong correlations at δ_C_/δ_H_ 111.4/6.95 (G_2_), 115.0/6.75 (G_5_), 119.9/6.85 (G_6_) for all hardwood and softwood lignins. Considerable amount of C_2,6_–H_2,6_ correlations in PB units were observed at δ_C_/δ_H_ 131.2/7.66 (PB_2,6_) for EOL–CW, EOL–BW, and EOL–AS, but not for EOL–EH and EOL–LP. This agreed well with the previous study in which no PB units were observed in eucalyptus lignin [33]. It is possible that the PB units could contribute to the high zeta potential of EOL–CW, EOL–BW, and EOL–AS, and give rise to the positive effect of these three lignins.

## Conclusions

Lignin inhibition or stimulation on enzymatic hydrolysis is mediated by hydrophobic interactions and electrostatic repulsions at the same time. Our results showed quantitatively that the lignin effect is a function of two factors. The inhibition is controlled by lignin hydrophobicity and the stimulation is governed by the negative zeta potential. The net effect of lignin depends on the combined influence of hydrophobicity and zeta potential. EOL–CW, EOL–BW, and EOL–AS showed high negative zeta potential (<−13 mV) and low hydrophobicity (0.4–0.8 L/g), which resulted in the positive effect on enzymatic hydrolysis. EOL–EH and EOL–LP showed low negative zeta potential (>−8 mV) and high hydrophobicity (0.8–1.1 L/g), which resulted in negative effect on enzymatic hydrolysis. A strong correlation was observed between the 72 h hydrolysis yield with hydrophobicity and zeta potential. Unexpectedly, the *p*-hydroxybenzoate lignin subunit (PB) was revealed by HSQC NMR in EOL–CW, EOL–BW, and EOL–AS, but not observed in EOL–EH and EOL–LP. We believe that the partial negative charge on the carbonyl oxygen in PB partially contributes to the negative potential of lignins. The results from this study have potential implications in biomass pretreatment, which could be very useful to increase lignin negative zeta potential and decrease hydrophobicity.

## Methods

### EOL lignins preparation and chemical composition analysis

Cottonwood, black willow, aspen, eucalyptus, and loblolly pine were collected by Forest Products Laboratory at Auburn University. Wood chips were ground with Waring pulverizer to the size of 1.0 × 1.0 × 0.3 cm (L × W × H).

Ethanol organosolv lignin (EOL) was collected from organosolv pretreatment of woody biomass as described previously [4]. Briefly, wood chips of cottonwood, black willow, aspen, and eucalyptus were pretreated in a 1.0 L Parr reactor with 65% (v/v) ethanol and 1% (w/w) sulfuric acid at 160 °C for 60 min, in a solid to liquid ratio of 1:7 (w/v). Loblolly pine was pretreated similarly, with 65% ethanol at 170 °C for 60 min. After pretreatment, the mixture was filtered and the liquid fraction was collected. Water was added into the spent liquid to precipitate the ethanol organosolv lignin.

The chemical composition of these lignins was determined using National Renewable Energy Laboratory (NERL) laboratory analytical procedures [34]. Neutral sugar composition of the lignins was determined by HPLC after two-step acid hydrolysis. Klason lignin was equivalent to the residue obtained after the acid hydrolysis. The polysaccharides and lignin contents in EOL preparations are summarized in Table 4.

**Table 4:** Chemical components of isolated lignins from organosolv-pretreated hydrolysate of cottonwood, black willow, aspen, eucalyptus, and loblolly pine

	Klason lignin (%)	ASL (%)	Glucan (%)	Xylan (%)	Mannan (%)
EOL–CW	91.0 ± 0.5	2.1 ± 0.01	ND	1.9 ± 0.02	ND
EOL–AS	92.1 ± 0.2	2.8 ± 0.02	ND	0.9 ± 0.0	0.4 ± 0.0
EOL–BW	91.2 ± 0.6	2.9 ± 0.02	ND	1.9 ± 0.01	ND
EOL–EH	93.5 ± 0.2	2.5 ± 0.01	ND	0.3 ± 0.0	ND
EOL–LP	98.1 ± 0.1	0.5 ± 0.02	ND	ND	ND

*ND* not detectable, *ASL* acid soluble lignin

### Cellulase enzymes and enzymatic hydrolysis of Avicel with addition of EOL lignins

A commercial cellulase, Cellic CTec2, was provided from Novozymes North America, Inc. (Franklinton, NC). The filter paper enzyme activity of Cellic CTec2 was 126 FPU/mL, and its protein content was 61 mg/mL. Cellulase C2730 was purchased from Sigma-Aldrich, Co. (St. Louis, MO). Microcrystalline cellulose, Avicel PH-101 was purchased from Sigma-Aldrich (St. Louis, MO).

Enzymatic hydrolysis processes of Avicel (98.72% glucan) or organosolv-pretreated aspen (OPAS) and loblolly pine (OPLP) were performed in 125 mL Erlenmeyer flasks with 50 mL of 50 mM citrate buffer at 2% glucan (w/v) as described previously. [21] Briefly, the hydrolysis of substrates with Cellic CTec2 was carried out at 50 °C and 150 rpm for 72 h. The enzyme loading was 5 FPU/g glucan for hydrolysis of Avicel or OPAS, and 10 FPU/g glucan for hydrolysis of OPLP. To examine the effect of EOL lignins on enzymatic hydrolysis, 4 g/L EOL lignins were added into the enzymatic hydrolysis system prior to the addition of cellulase enzyme. To measure the hydrolysis yield of pure and pretreated biomass, the samples were taken from the hydrolysis solution at various time intervals. The glucose content was determined by HPLC with Aminex HPX-87P column. Nanopure water was used for mobile phase at a flow rate of 0.6 mL/min. The hydrolysis yield of Avicel was calculated from the released glucose, as a percentage of the theoretical glucose available in Avicel. The free enzyme concentration in supernatant was determined by Bradford assay, and presented in the percentage of the total protein concentration. All hydrolysis experiments were run in duplicates.

### Cellulase adsorption isotherms

Cellulase adsorption experiments were performed in 25 mL Erlenmeyer flasks containing 2% (w/v) EOL lignins in 5 mL of 0.05 M citrate buffer (pH 4.8). Cellulase C2730 with various concentrations (0.01–0.4 mg/mL) was added in the flasks and incubated with EOL lignins at 4 °C and 150 rpm for 3 h. After reaching equilibrium, the suspension was separated by centrifugation and the supernatant was collected for the analysis of free enzyme. Free enzyme protein in the supernatant was quantified by Bradford assay [35]. The adsorbed enzyme on lignin samples was calculated from the difference between the initial enzyme dosage and the free enzyme content. Langmuir adsorption isotherm (Eq. 1) was fitted to the adsorption data.

1 $$\varGamma = \frac{{\varGamma_{ \text{max} } {\text{KC}}}}{{ 1{\mkern 1mu} { + }{\mkern 1mu} {\text{KC}}}},$$

where *C* is the concentration of free enzyme protein in solution (mg/mL), *Γ* is the amount of adsorbed protein (mg/g of lignin), *Γ*_max_ is the maximal adsorbed protein (mg/g of lignin), and *K* is the Langmuir constant (mL/mg of protein).

### Determination of lignin hydrophobicity, zeta potential, surface morphology, and particle size

Surface hydrophobicity of organosolv lignins was quantified by measuring the adsorption of Rose Bengal [36]. Briefly, various concentrations of lignin (2–10 g/L) was mixed with 40 mg/L Rose Bengal in 50 mM citrate buffer (pH 4.8) and incubated at 50 °C, 150 rpm for 2 h. Rose Bengal distributes between the aqueous phase and the lignin surface. These phases were separated by centrifugation. The free dye content in the supernatant was determined by measuring the adsorption at 543 nm using a UV–Vis spectrometer. The adsorbed dye on the lignin surface was calculated by the difference between the initial dye content and the free dye content. The partitioning quotient (PQ) was calculated as PQ = amount Rose Bengal bound on surface/amount Rose Bengal in dispersion medium. PQ was plotted against the lignin content. The slopes of the straight lines were regarded as a measure of the surface hydrophobicity of lignin (L/g). The zeta potentials of the lignin samples were measured by a Zetasizer (Nano-ZS, Malvern Instruments Ltd, Worcestershire, UK) with laser Doppler microelectrophoresis after blending 1 mg lignin with 1 mL of 50 mM citrate buffer and dispersing using an ultrasonic disperser. The measurements were repeated for three times and the results were analyzed by Dispersion Technology Software (DTS).

The surface of EOL lignins was analyzed by a field emission scanning electron microscopy (JEOL 7000F) operated at 20.0 kV. Lignin samples were coated with a thin gold layer (50 nm) using PELCO SC-6 Sputter Coater. The particle size was calculated using software ImageJ.

### NMR spectroscopy analysis of the organosolv lignins

NMR spectra were acquired on a Bruker (Billerica, MA) 600 MHz spectrometer at 25 °C. The organosolv lignins (60 mg) were dissolved in 0.5 mL of DMSO-d_6_ (0.6 mL) and chromium(III) acetylacetonate (20 µL, 0.01 M) for acquiring the quantitative 13C NMR spectra. Operating conditions were listed as below: 90° pulse angle, 0.9 s acquisition time, and 1.8 s relaxation delay with a total of 20,000 scans per sample.

For 2D HSQC NMR spectra, EOL lignins (60 mg) were dissolved in 0.5 mL of DMSO-d_6_. The central DMSO solvent peak was used as an internal reference for all samples (δ_C_ 39.5, δ_H_ 2.49 ppm). HSQC spectra were accomplished using the Bruker pulse program “hsqcetgp” and had following parameters: the ^1^H dimension (F_2_) was acquired from 10 to 0 ppm with 4096 data points, the ^13^C dimension (F_1_) was obtained from 200 to 0 ppm with 64 scans, and 256 increments. Acquisition time of 0.24 and 0.028 s were used for ^1^H and ^13^C, respectively. The total acquisition time was 17.5 h. Afterwards, Fourier transformation and phase correction were applied in both dimensions on spectra with Topspin 2.1.

## Additional file

**Additional file 1: Table S1.** Assignments of ^13^C–^1^H cross-signals in 2D HSQC spectra of EOL lignins. **Figure S1.** Effect of different concentration of added lignin on enzymatic hydrolysis of Avicel. **Figure S2.** SEM images of EOL lignins. **Figure S3.** Particle size distribution of EOL lignins. **Figure S4.** Main structures of EOL lignins: (A) β-aryl-ether units (β-*O*-4); (B) resinol substructures (β–β); (C) phenylcoumaran substructures (β-5); (G) guaiacyl units; (S) syringyl units; (S′) oxidized syringyl units bearing a carbonyl at C_*α*_; (PB) *p*-hydroxybenzoate units.

## Abbreviations

EOL: ethanol organosolv lignin
CW: cottonwood
BW: black willow
AS: aspen
EH: eucalyptus
LP: loblolly pine
OPAS: organosolv-pretreated aspen
OPLP: organosolv-pretreated loblolly pine
DMSO-d6: deuterated dimethyl sulfoxide
SEM: scanning electron microscopy
FT-IR: fourier transform infrared
NREL: National Renewable Energy Laboratory
HPLC: high-performance liquid chromatography
2D HSQC NMR: two-dimensional heteronuclear single quantum coherence nuclear magnetic resonance
S: syringyl
S′: oxidized syringyl
G: guaiacyl
PB: *p*-hydroxybenzoate subunit
