# Supplementary material for: Stimulation and inhibition of enzymatic hydrolysis by organosolv lignins as determined by zeta potential and hydrophobicity
Source: Biotechnol Biofuels. 2017 Jun 24;10:162. doi: 10.1186/s13068-017-0853-6 (PMC5483266; doi:10.1186/s13068-017-0853-6)
Supplement: Supplementary file 1 — Additional file 1: Table S1. Assignments of 13C–1H cross-signals in 2D HSQC spectra of EOL lignins. Figure S1. Effect of different concentration of added lignin on enzymatic hydrolysis of Avicel. Figure S2. SEM images of EOL lignins. Figure S3. Particle size distribution of EOL lignins. Figure S4. Main structures of EOL lignins: (A) β-aryl-ether units (β-O-4); (B) resinol substructures (β–β); (C) phenylcoumaran substructures (β-5); (G) guaiacyl units; (S) syringyl units; (S′) oxidized syringyl units bearing a carbonyl at Cα; (PB) p-hydroxybenzoate units. [file 13068_2017_853_MOESM1_ESM.docx]

**Stimulation and inhibition of enzymatic hydrolysis by organosolv lignins as determined by zeta potential and hydrophobicity**

Yang Huang^1^, Shaolong Sun^2^, Chen Huang^1^, Qiang Yong^1,*^, Thomas Elder^3^, Maobing Tu^2,*^

^1^College of Chemical Engineering, Nanjing Forestry University, Nanjing 210037, China

^2^Department of Biomedical, Chemical and Environmental Engineering, University of Cincinnati, 2901 Woodside Drive, Cincinnati, Ohio, 45221, United States.

^3^USDA-Forest Service, Southern Research Station, 521 Devall Drive, Auburn, Alabama 36849, United States

* Corresponding author

Email address: [tumg@uc.edu](mailto:tumg@uc.edu) or swhx@njfu.com.cn

Tel: +1 513 556 2259; or +86 25 85427702

**Table S1:** Assignments of ^13^C-^1^H cross-signals in 2D-HSQC NMR spectra of EOL lignins

**Figure S1:** Effect of different concentration of added lignin on enzymatic hydrolysis of Avicel

**Figure S2:** SEM images of EOL lignins

**Figure S3**: Particle size distribution of EOL lignins

**Figure S4:** Main structures of EOL lignins: (A) *β*-aryl-ether units (*β*-*O*-4); (B) resinol substructures (*β*-*β*); (C) phenylcoumaran substructures (*β*-5); (G) guaiacyl units; (S) syringyl units; (S′) oxidized syringyl units bearing a carbonyl at C*_α_*; (PB) *p*-Hydroxybenzoate units.

**Table S1** Assignments of ^13^C-^1^H cross-signals in HSQC spectra of EOL lignins

| Lables | *δ*_C_/*δ*_H_ (ppm) | Assignments |
| --- | --- | --- |
| C*_β_* | 53.0/3.45 | C*_β_*−H*_β_* in phenylcoumaran substructures (C) |
| B*_β_* | 53.5/3.04 | C*_β_*−H*_β_*in *β*-*β* (resinol) substructures (B) |
| −OCH_3_ | 55.7/3.73 | C−H in methoxyls |
| A*_γ_* | 59.8/3.60 | C*_γ_*−H*_γ_* in *β*-*O*-4 substructures (A) |
| C*_γ_* | 62.7/3.71 | C*_γ_*−H*_γ_*in phenylcoumaran substructures (C) |
| A′*_α_*-OEt | 63.7/3.33 | C−H in *α*-OEt*β*-*O*-4′ substructures (A′) |
| B*_γ_* | 71.2/3.82−4.18 | C*_γ_*−H*_γ_*in *β*-*β*resinol substructures (B) |
| A*_α_* | 71.9/4.88 | C*_α_*−H*_α_* in *β*-*O*-4 substructures (A) |
| E*_α_* | 78.3/5.57 | C*_α_*−H*_α_* in *α*,-*β*-diaryl ethers (E) |
| A′*_α_* | 79.9/4.50 | C*_α_*−H*_α_* in *α*-ethoxylated *β*-*O*-4 substructures (A′) |
| A*_β_*_(G)_ | 83.3/4.29 | C*_β_*−H*_β_* in *β*-*O*-4 linked to G unit (A) |
| A′*_β_*_(G)_ | 82.2/4.39 | C*_β_*−H*_β_* in *β*-*O*-4 linked to G unit (A′) |
| B*_α_* | 85.1/4.65 | C*_α_*−H*_α_* in *β*-*β* resinol substructures (B) |
| A*_β_*_(S)_ | 86.1/4.14 | C*_β_*−H*_β_*in *β*-*O*-4 linked to a S unit (A) |
| A′*_β_*_(S)_ | 84.7/4.20 | C*_β_*−H*_β_*in *β*-*O*-4 linked to a S unit (A′) |
| C*_α_* | 87.2/5.48 | C*_α_*−H*_α_*in phenylcoumaran substructures (C) |
| S_2,6_ | 103.7/6.68 | C_2,6_−H_2, 6_ in syringyl units (S) |
| S′_2,6_ | 106.5/7.33 | C_2,6_−H_2,6_ in oxidized S units (S′) |
| G_2_ | 111.4/6.95 | C_2_−H_2_ in guaiacyl units (G) |
| G_5_ | 115.0/6.75 | C_5_−H_5_ in guaiacyl units (G) |
| G_6_ | 119.9/6.85 | C_6_−H_6_ in guaiacyl units (G) |
| PB_2,6_ | 131.3/7.62 | C_2,6_−H_2, 6_ in *p*-hydroxybenzoate substructures (PB) |

**Fig.S1** Effect of different concentration of added lignin on enzymatic hydrolysis of Avicel


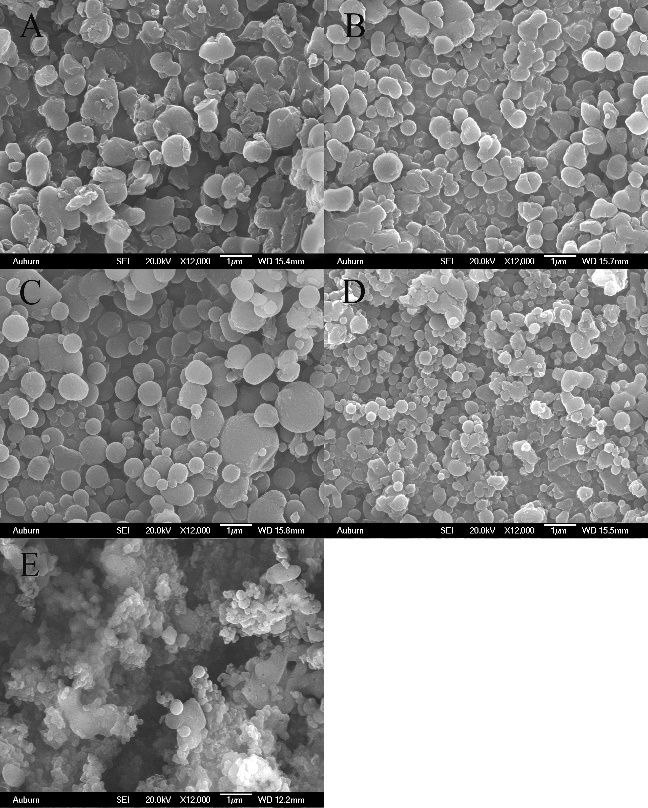


**Fig.S2** Scanning electron microscopy (SEM) images of EOL lignins. Lignin preparations were obtained from organosolv pretreated aspen (A), black willow (B), cottonwood (C), eucalyptus (D) and loblolly pine (E).

| A | B |
| --- | --- |
| C | D |
| E |  |
| **Fig.S3** Particle size distribution of isolated lignins. Lignin preparations were obtained from organosolv pretreated aspen (A), black willow (B), cottonwood (C), eucalyptus (D) and loblolly pine (E). | |


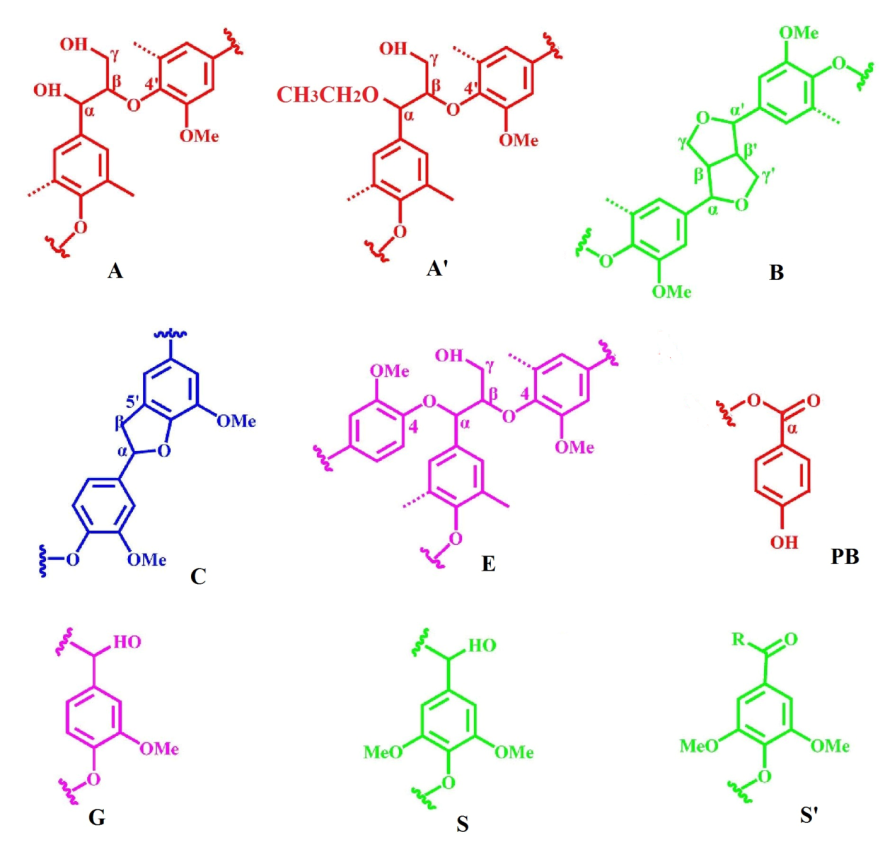


**Fig.S4** Main structures in the organosolv lignins: (A) *β*-aryl-ether units (*β*-*O*-4); (B) resinol substructures (*β*-*β*); (C) phenylcoumaran substructures (*β*-5); (G) guaiacyl units; (S) syringyl units; (S′) oxidized syringyl units bearing a carbonyl at C*_α_*; (PB) *p*-Hydroxybenzoate units.
